# Evaluate the Effect of Commercially Available Denture Cleansers on Surface Hardness and Roughness of Denture Liners at Various Time Intervals

**Published:** 2016-12

**Authors:** Hilal S. Mohammed, Sumeet Singh, Prasad A. Hari, G. S. Amarnath, Vinaya Kundapur, Naveed Pasha, M. Anand

**Affiliations:** 1 Reader, Department of prosthodontics, M R Ambedkar Demtal college & Hospital, Bangalore, INDIA;; 2 Post graduate student, Department of prosthodontics, M R Ambedkar Demtal college & Hospital, Bangalore, INDIA; 3 Reader, Department of prosthodontics, M R Ambedkar Dental College & Hospital, Bangalore, INDIA; 4 Professor & HOD, Department of Prosthodontics, M R Ambedkar Dental College & Hospital, Bangalore, INDIA; 5 Senior Lecturer, Departemenrt of prosthodontics, M R Ambedkar Dental College & Hospital, Bangalore, INDIA; 6 Post graduate student, Department of prosthodontics, M R Ambedkar Demtal college & Hospital, Bangalore, INDIA; 7 Reader, department of prosthodontics, M R Ambedkar Dental College & Hospital, Bangalore, INDIA

**Keywords:** Acrylic liner, Silicon liner, Denture cleanser, Surface hardness, Surface roughness

## Abstract

**Background and objective:**

Chemical cleansing by denture cleansers is first choice for denture plaque control. The most common problems while using denture cleansers are hardening, porosity, odor sorption, water sorption, solubility, and colour change, bacterial and fungal growth. Chemical cleansing procedures have been found to have an effect on the physical and mechanical properties of denture liners. Thus, this study was conducted to evaluate the effect of commercially available denture cleansers on surface hardness and roughness of acrylic and silicon based denture liners at various time interval.

**Method:**

Two autopolymerising denture liners Kooliner (acrylic) and GC reline soft (silicon) were tested with two commercially available denture cleansers, polident and efferdent plus. Total of 120 specimens were prepared and all the specimens were divided into six groups based on the relining materials and denture cleansers used. Surface hardness and surface roughness was tested using Shore A durometer and profilometer respectively at the end of day 1, day 7, day 30 and day 90. All the specimens were stored in artificial saliva throughout the study. Cleanser solution was prepared daily by adding Polident and Efferdent plus denture cleanser tablet into 250ml of enough very warm (not hot) water. Acrylic and silicon liner groups were cleansed in a solution of denture cleanser and water for 15 minutes daily, rinsed with water and stored in artificial saliva at room temperature. The data was analyzed with one way ANOVA and independent t-test.

**Result:**

The acrylic soft lining showed gradual hardening and increase in surface roughness after immersion in denture cleanser and also with time. Acrylic liner material showed maximum hardness and roughness with Polident followed by Efferdent plus and water (control group). Silicone lining material showed a slight difference in hardness and roughness between the test group and control group. There was a slight increase in hardness in all the groups with time. Very slight increase in mean surface roughness of all the silicon liner groups from day 1 to day 90 was observed. A statistically significant change was noted between and within the all silicon liner groups on day 7, day 30 and day 90.

**Conclusion:**

The average surface hardness and surface roughness were lower in silicon liner material than acrylic liner material. Maximum surface roughness was noted by Polident followed by Efferdent Plus and Water for both acrylic liner group and silicon liner group.

## INTRODUCTION

The accuracy of denture fit is an important factor in the retention of denture. The use of resilient lining materials are useful in removable prosthodontics because of their capability of restoring health to inflamed mucosa, leading to more equal distribution of functional load on the denture foundation area and improving the fitting denture surface and retention of the prosthesis (1). Soft denture lining materials have been used in dentistry for more than a century, with earliest soft liner being natural rubbers. One of the first synthetic resins developed in 1945 as a soft liner was a plasticized poly vinyl resin followed by the introduction of silicones in 1958 (2).

The resilient denture lining materials can be divided into two types: plasticized acrylic resins and silicone elastomers. Acrylic resin-based resilient denture liners often contain plasticizers that may leach out of the material, resulting in hardening of the liners with time. For silicone-based resilient lining materials, the polymer is an elastomer, which does not require an external plasticizer and is therefore, more stable over time. Resilient reline materials are available in autopolymerizing and heat polymerizing forms. Autopolymerizing relining materials can be an attractive alternative to heat-polymerized liners because they can be placed chairside, are easier to apply, and require no laboratory procedures (3). Rigid auto-polymerizing acrylic resin is used to reline dentures directly in the mouth. The resin is polymerized by mixing a powder of poly (methyl methacrylate) with aliquid of methyl methacrylate and using peroxides, amines, and plasticizers to control the chemical process. The effect of this chemical reaction on the oral mucosa are unknown; however, many patients find the procedure uncomfortable and distasteful (4). The use soft denture liners has become increasingly popular for providing comfort for denture wearers. Soft denture liners are often used for patients who cannot tolerate a conventional denture base (5).

Success of dentures made from two different materials depends on adequate bond between the materials. Hence, reason for failure of soft lined denture is structural differences between the materials. Hardness of denture liners is most important, with a direct impact on malleability, ductibility and abrasion resistance. Surface roughness is also important property of a denture liner, as rough denture surface leads to biofilm formation and colonization by Candida albicans (6). Routine dentures cleaning not only removes plaque and prevent re-accumulation plaque, it also removes mucin, calculus, food debris, calculus, and exogenous discoloration (7).

Denture plaque control using mechanical and chemical methods is essential for maintenance of good oral hygiene of denture wearers. However, mechanical cleansing is not advisable for soft denture liners since it can damage resilient lining. Chemical cleansing by denture cleansers is first choice for denture plaque control (8). The most common problems while using denture cleansers are hardening, porosity, odor sorption, water sorption, solubility, and colour change, bacterial and fungal growth (9).

Although chemical cleansing has been considered an efficacious method to prevent Candida albicans invasion and denture plaque formation, some types of denture cleansers have been reported to cause significant deterioration of tissue conditioners in a relatively short time. A roughened surface facilitates colonization by microorganisms. Therefore denture cleansers used for plaque control of tissue conditioners should reduce microbial contamination and have a minimum effect on physical properties of liner (10).

The most common problems encountered while using soft denture liners are water sorption and solubility. In use, they are constantly bathed in saliva, and when out of the mouth, they are usually immersed either solution or denture cleansers or water for storage. During such immersion, soft lining materials undergo two responses: plasticizers and other soluble components are leached out and water or saliva is absorbed. So, an ideal processed soft liner should have no soluble components and low water sorption (11).

As per the data acquired through studies conducted, hygiene procedures have been found to have an effect on the physical and mechanical properties of denture liners because they can cause loss of plasticizers and soluble component, or water absorption or absorption of saliva by the denture lining materials. Thus, the aim of this study is to evaluate the effect of commercially available denture cleansers on surface hardness and surface roughness of various denture liners.

The aim and objective of this study was:

1. To determine the influence of denture cleansers on surface hardness and surface roughness of denture liners.
2. To compare the efficiency of commercially available denture cleansers on denture liners.

## MATERIALS & METHODS

A total of hundred and twenty cylindrical specimens were made using custom made metal mould with the dimension of 15 mm in diameter and 10 mm in height (according to ASTM: D -2240 64T).

### Preparation of kooliner specimens

Total sixty kooliner specimens were made of dimension of 15 mm × 10 mm with the help of custom made metal mould. Petroleum jelly was applied on the mould for easy removal of the specimens. Base of the mould was placed on a glass slab covered with cellophane sheet to facilitate separation of mould from the glass slab. Both the relining materials were manipulated according to manufacturer instructions. Recommended power/liquid ratio for kooliner is 15 ml powder to 6ml liquid. Pour liquid into the mixing cup and then add the powder slowly. Stir thoroughly for no more than 30 seconds and avoid the introduction of air bubble. After approximately 1-2 minutes expressed the mix into the mould. The mould was then covered with cellophane sheet and another glass slab was pressed tightly against the mould to remove the excess material and to shape the specimens according to the dimensions of the mould. When curing was complete (10 minutes), specimens were removed from the mould and excess was trimmed.

### Preparation of GC reline soft specimens

Total sixty GC reline soft specimens were made of dimension of 15 × 10 mm with the help of custom made metal mould. Petroleum jelly was applied on the mould for easy removal of the specimens. Base of the mould was placed on a glass slab covered with cellophane sheet to facilitate separation of mould from glass slab. GC reline soft cartridge was attached to the cartridge dispenser, cartridge cap was replaced with mixing tip and material was directly expressed into the metal mould.

The mould was then covered with cellophane sheet and another glass slab was pressed tightly against the mould to remove the excess material and to shape the specimens according to the dimensions of the mould. After setting time of 4 minutes of the material, specimens were removed from the mould and using a B.P. blade, excess material on the edge was removed. Cleanser solution was prepared daily by adding Polident or Efferdent plus denture cleanser tablet into 250ml of enough very warm (not hot) water.

### Storage and cleansing of specimens

All the specimens were divided into six groups based on the relining materials and denture cleansers used and each group was tested at a time interval of day 1, day 7, day 30 and day 90. All the specimens were stored in artificial saliva throughout the study. Specimens of group AP (Acrylic specimen cleansed daily in Polident denture cleanser) and SP(Silicon specimen cleansed daily in Polident denture cleanser) were cleansed in a solution of Polident denture cleanser solution for 15 minutes daily, rinsed in water and stored in artificial saliva at room temperature. Specimens of group AE (Acrylic specimen cleansed daily in Efferdent plus denture cleanser) and SE (Silicon specimen cleansed daily in Efferdent denture cleanser) were cleansed daily in a solution of Efferdent denture cleanser solution for 15 minutes, rinsed in water and stored in artificial saliva at room temperature. Specimens of group AW (Acrylic specimen cleansed daily in Water) and SW (Silicon specimen cleansed daily in Water) were cleansed daily on water and stored in artificial saliva at room temperature.

### Total of 120 specimens were prepared and divided into 6 groups

Kooliner specimen cleansed daily in Polident denture cleanser-20;

Kooliner specimen cleansed daily in Efferdent denture cleanser-20;

Kooliner specimen cleansed daily in water-20;

GC reline soft specimen cleansed daily in Polident denture cleanser-20;

GC reline soft specimen cleansed daily in Efferdent denture cleanser-20;

GC reline soft specimen cleansed daily in water -20.

### Testing of specimens

Specimens were subjected to surface hardness and surface roughness testing at time intervals of day 1, day 7, day 30 and day 90. Hardness was measured using Shore A durometer and surface roughness was measured using Profilometer.

### Statistical Methods Employed

Mean, Standard deviation, Independent sample t-test and ANOVA were the statistical methods employed in the study. ANOVA was performed across mean of the specimen group for each series of test when significant statistical difference was detected. Independent sample t-test at the significance level of 0.05 was applied to the groups to determine statistical difference between two means.

## RESULTS

Statistical analysis, as given in Table 1, showed that on day 1 both the test (AP and AE) and control (AW)acrylic liner groups showed no difference in hardness but there was significant (*P*<0.05) increase in hardness was noted between and within the all acrylic liner groups on day 7, day 30 and day 90. The highest mean hardness was observed in AP acrylic liner group followed by AE and AW acrylic liner group (Graph 1).

**Table 1:** One way analysis of variance for mean surface hardness of acrylic liner groups

**(a) Descriptive:-**						
**Shore A Hardness**	**Cleanser**	**n**	**Mean**		**Std. Deviation**	

Day 1	P	20	16.0000		0.0000	
	E	20	16.2000		0.7677	
	W	20	16.0000		0.7947	
Day 7	P	20	19.8000		1.0052	
	E	20	18.9000		0.8522	
	W	20	17.1000		0.7181	
Day 30	P	20	31.3000		1.4545	
	E	20	28.6000		0.8207	
	W	20	22.7000		0.9233	
Day 90	P	20	46.1000		0.7181	
	E	20	42.9000		1.6827	
	W	20	40.1000		0.8522	

**(b) ANOVA:-**						
**Shore A Hardness**	**Source Of Variation**	**Sum Of Squares**	**df**	**Mean Square**	**F**	**Sig. (P)**

Day 1	Between groups	0.533	2	0.267	0.655	0.523
	Within groups	23.200	57	0.407		
Day 7	Between groups	75.600	2	37.800	50.341	0.000
	Within groups	42.800	57	0.751		
Day 30	Between groups	773.733	2	386.867	318.662	0.000
	Within groups	69.200	57	1.214		
Day 90	Between groups	360.533	2	180.267	132.755	0.000
	Within groups	77.400	57	1.358		

Table 2 shows that there was an increase in mean surface roughness (Ra) from day 1 to day 90 of both test (AP and AE) and control (AW) acrylic liner groups but a statistically significant (*P*<0.05) increase in surface roughness was noted between and within the all acrylic liner on day 7, day 30 and day 90. Highest mean surface roughness was observed in AP acrylic liner group followed by AE and AW acrylic liner group (Graph 2).

**Table 2:** One way analysis of variance for mean surface roughness of acrylic liner groups

**(a) Descriptive:-**						
**Surface Roughness**	**Cleanser**	**n**	**Mean**		**Std.**	

Day 1	P	20	1.6575		0.0120	
	E	20	1.6529		0.0108	
	W	20	1.6531		0.0077	
Day 7	P	20	1.9839		0.0106	
	E	20	1.7577		0.0081	
	W	20	1.6878		0.0121	
Day 30	P	20	2.1350		0.0098	
	E	20	1.9142		0.0045	
	W	20	1.8229		0.0048	
Day 90	P	20	2.5195		0.0060	
	E	20	2.1875		0.0103	
	W	20	2.1091		0.0139	

**(b) ANOVA:-**						
**Surface Roughness**	**Source of Variation **	**Sum of Squares **	**df**	**Mean Square **	**F**	**Sig. (P)**

Day 1	Between groups	0.000	2	0.000	1.257	0.292
	Within groups	0.006	57	0.000		
Day 7	Between groups	0.958	2	0.479	4417.954	0.000
	Within groups	0.006	57	0.000		
Day 30	Between groups	1.030	2	0.515	10916.326	0.000
	Within groups	0.003	57	0.000		
Day 90	Between groups	1.899	2	0.949	8423.637	0.000
	Within groups	0.006	57	0.000		

Table 3 shows that there was a slight increase in hardness of all the silicon liner groups (SP, SE and SW) from day 1 to day 90 but a statistically significant (*P*<0.05) change was noted between and within the all silicon liner groups (SP, SE and SW) on day 7, day 30 and day 90. The highest mean hardness was observed in SP silicon liner group followed by SE and SW silicon liner group (Graph 3).

**Table 3:** One way analysis of variance for mean surface hardness of silicon liner groups

**(a) Descriptive:-**						
**Shore A Hardness **	**Cleanser **	**n **	**mean **		**Std. Deviation **	

Day 1	P	20	20.3000		0.6560	
	E	20	20.3000		0.6569	
	W	20	20.1000		0.7181	
Day 7	P	20	22.8000		1.1050	
	E	20	22.1000		0.7181	
	W	20	20.5000		1.0513	
Day 30	P	20	25.1000		1.5525	
	E	20	23.2000		0.6155	
	W	20	22.0000		1.2139	
Day 90	P	20	28.0000		0.9176	
	E	20	25.6000		0.9403	
	W	20	24.2000		0.8944	

**(b) ANOVA:-**						
**Shore A Hardness**	**Source of Variation**	**Sum of Squares**	**df**	**Mean Square **	**F**	**Sig. (P)**

Day 1	Between Groups	0.533	2	0.267	0.580	0.563
	Within Groups	26.200	57	0.460		
Day 7	Between Groups	55.600	2	27.800	29.344	0.000
	Within Groups	54.000	57	0.947		
Day 30	Between Groups	97.73	2	48.867	34.388	0.000
	Within Groups	81.000	57	1.421		
Day 90	Between Groups	147.733	2	73.867	87.717	0.000
	Within Groups	48.000	57	0.842		

Table 4 shows that there was very slight increase in mean surface roughness (Ra) of all the silicon liner groups from day 1 to day 90 and no significant (*P*>0.05) change in surface roughness was noted between and within the groups of the test (SP and SE) and control (AW) silicon liner groups on day 1 but a statistically significant (*P*<0.05) change was noted between and within the all silicon liner groups (SP, SE and SW) on day 7, day 30 and day 90. The highest mean roughness was observed in SP silicon liner group followed by SE and SW silicon liner group (Graph 4).

**Table 4:** One way analysis of variance for mean surface roughness of silicon liner groups

**(a) Descriptive:-**						
**Surface Roughness**	**Cleanser**	**n**	**Mean**		**Std. Deviation**	

Day 1	P	20	1.3226		0.0092	
	E	20	1.3217		0.0083	
	W	20	1.3218		0.0111	
Day 7	P	20	1.3543		0.0077	
	E	20	1.3405		0.0079	
	W	20	1.3335		0.0096	
Day 30	P	20	1.3803		0.0068	
	E	20	1.3711		0.0028	
	W	20	1.3430		0.0092	
Day 90	P	20	1.4131		0.0061	
	E	20	1.3832		0.0044	
	W	20	1.3532		0.0068	

**(b) ANOVA:-**						
**Surface Roughness**	**Source of Variation **	**Sum of Squares **	**df**	**Mean square**	**F**	**Sig. (P) **

Day 1	Between groups	0.000	2	0.000	0.052	0.949
	Within groups	0.005	57	0.000		
Day 7	Between groups	0.004	2	0.002	30.947	0.000
	Within groups	0.004	57	0.000		
Day 30	Between groups	0.015	2	0.008	161.218	0.000
	Within groups	0.003	57	0.000		
Day 90	Between groups	0.036	2	0.018	518.499	0.000
	Within groups	0.002	57	0.000		

Table 5 shows that Acrylic liner group (AP) showed higher surface hardness than silicon liner group (SP) when treated with cleanser P. This difference was statistically significant at all time intervals (Graph 5).

**Table 5:** Comparison of mean surface hardness between acrylic and silicon liner groups when treated with cleanser P at all time interval (AP, SP)

**(a) Group Statics:-**				
	**Hardness**	**n **	**Mean **	**Std. Deviation **

Day 1	AH	20	16.000	0.0000
	SH	20	20.300	0.6569
Day 7	AH	20	19.800	1.0052
	SH	20	22.800	1.1050
Day 30	AH	20	31.300	1.4545
	SH	20	25.100	1.5525
Day 90	AH	20	46.100	0.7181
	SH	20	28.000	0.9176

**(b) Independent sample Test:-**				
**Shore A Hardness**	**t**	**df**	**Sig. (2-tailed) **	**Mean difference**

Day 1	-29.272	38	0.000	-4.3000
Day 7	-8.981	38	0.000	-3.0000
Day 30	13.033	38	0.000	6.2000
Day 90	69.464	38	0.000	18.100

Table 6 shows that Acrylic liner group (AE) showed higher surface hardness than silicon liner group (SE) when treated with cleanser E. This difference was statistically significant at all time intervals (Graph 6).

**Table 6:** Comparison of mean surface hardness between acrylic and silicon liner groups when treated with cleanser E at all time interval (AE, SE)

**(a) Group Statics:- **				
	**Hardness**	**n **	**Mean **	**Std. Deviation**

Day 1	AH	20	16.200	0.7677
	SH	20	20.300	0.6569
Day 7	AH	20	18.900	0.8522
	SH	20	22.100	0.7181
Day 30	AH	20	28.600	0.8207
	SH	20	23.200	0.6155
Day 90	AH	20	42.900	1.6827
	SH	20	25.600	0.9403

**(b) Independent Sample test:-**				
**Shore A Hardness**	**t-test for Equality of Means**			
	**t**	**df **	**Sig. (2-tailed)**	**Mean difference**

Day 1	-18.146	38	0.000	-4.1000
Day 7	-12.841	38	0.000	-3.2000
Day 30	23.538	38	0.000	5.4000
Day 90	40.136	38	0.000	17.3000

Table 7 shows that Acrylic liner group (AW) showed higher surface hardness than silicon liner group (AW) when treated with cleanser W. This difference was statistically significant at all time intervals (Graph 7).

**Table 7:** Comparison of mean surface hardness between acrylic and silicon liner groups when treated with cleanser W at all time interval (AW, SW)

**(a) Group Statics:-**				
	**Hardness **	**n**	**Mean**	**Std. Deviation**

Day 1	AH	20	16.000	0.7947
	SH	20	20.100	0.7181
Day 7	AH	20	17.100	0.7181
	SH	20	20.500	1.0513
Day 30	AH	20	22.700	0.9233
	SH	20	22.000	1.2139
Day 90	AH	20	40.100	0.8522
	SH	20	24.200	0.8944

**(b) Independent Sample Test:-**				
**Shore A Hardness**	**t-test for Equality of Means**			
	**t **	**df **	**Sig. (2-tailed)**	**Mean difference**

Day 1	-17.118	38	0.000	-4.1000
Day 7	-11.943	38	0.000	-3.4000
Day 30	2.052	38	0.047	0.7000
	57.556	38	0.000	15.9000

Table 8 shows that Acrylic liner group (AP) showed higher surface roughness than silicon liner group (SP) when treated with cleanser P. This difference was statistically significant at all time intervals (Graph 8).

**Table 8:** Comparison of mean surface roughness between acrylic and silicon liner groups when treated with cleanser P at all time interval (AP, SP)

**(a) Group Statics:-**				
	**Roughness**	**n **	**Mean **	**Std. Deviation**

Day 1	AH	20	1.6575	0.0120
	SH	20	1.3226	0.0092
Day 7	AH	20	1.9839	0.0106
	SH	20	1.3543	0.0077
Day 30	AH	20	2.1350	0.0098
	SH	20	1.3803	0.0068
Day 90	AH	20	2.5195	0.0060
	SH	20	1.4131	0.0061

**(b) Independent sample Test:-**				
**Surface Roughness**	**t-test for Equality of Means**			
	**t **	**df **	**Sig.( 2-tailed) **	**Mean difference**

Day 1	98.534	38	0.000	0.3349
Day 7	213.973	38	0.000	0.6296
Day 30	281.869	38	0.000	0.7547
Day 90	570.982	38	0.000	1.1064

Table 9 shows that Acrylic liner group (AE) showed higher surface roughness than silicon liner group (SE) when treated with cleanser E. This difference was statistically significant at all time intervals (Graph 9).

**Table 9:** Comparison of mean surface roughness between acrylic and silicon liner groups when treated with cleanser E at all time interval (AE, SE)

**(a) Group Statics:-**				
	**Roughness **	**n **	**Mean**	**Std. Deviation**

Day 1	AH	20	1.6525	0.0109
	SH	20	1.3217	0.0083
Day 7	AH	20	1.7685	0.0508
	SH	20	1.3405	0.0079
Day 30	AH	20	1.9246	0.0476
	SH	20	1.3711	0.0028
Day 90	AH	20	2.2035	0.0731
	SH	20	1.3832	0.0044

**(b) Independent Sample test:-**				
**Surface Roughness**	**t-test for Equality of Means**			
	**t **		**Sig. ( 2-tailed) **	**Mean difference**

Day 1	107.457	38	0.000	0.3308
Day 7	37.176	38	0.000	0.4280
Day 30	51.904	38	0.000	0.5535
Day 90	50.090	38	0.000	0.8203

Table 10 shows that Acrylic liner group (AW) showed higher surface roughness than silicon liner group (SW) when treated with cleanser W. This difference was statistically significant at all time intervals (Graph 10).

**Table 10:** Comparison of mean surface roughness between acrylic and silicon liner groups when treated with cleanser W at all time interval (AW, SW)

**(a) Group Statics:-**				
	**Roughness**	**n**	**Mean**	**Std. Deviation**

Day 1	AH	20	1.6531	0.0077
	SH	20	1.3218	0.0111
Day 7	AH	20	1.6878	0.0121
	SH	20	1.3335	0.0096
Day 30	AH	20	1.8229	0.0048
	SH	20	1.3430	0.0092
Day 90	AH	20	2.1091	0.0139
	SH	20	1.3532	0.0068

**(b) Independent Sample Test:-**				
**Surface Roughness**	**t-test for Equality of Means**			
	**t**	**df **	**Sig. ( 2-tailed)**	**Mean difference**

Day 1	109.211	38	0.000	0.3313
Day 7	102.275	38	0.000	0.3543
Day 30	205.033	38	0.000	0.4799
Day 90	217.804	38	0.000	0.7559

## DISCUSSION

The accuracy of denture fit is an important factor in the retention of denture. However, the process of alveolar resorption is irreversible and may lead to inadequate fit of prosthesis. The use of resilient lining materials is useful in removable prosthodontics because of their capability of restoring health of inflamed mucosa (1).

Liners are made-up of materials from several chemical families. These materials undergo chemical changes over time as patients use dentures in either the aqueous environment of their mouth or, if not in use, then in tap water or denture cleansers. The most common problems while using denture cleansers are hardening, porosity, odor sorption, water sorption, solubility, and color change, bacterial and fungal growth.6 Hardness of denture liner is important, with a direct impact on malleability, ductibility and abrasion resistance. Surface roughness is also important property of a denture liner, as rough denture surface leads to biofilm formation and colonization of Candida albicans (7).

Denture plaque control using mechanical and chemical methods is essential for maintenance of good oral hygiene of denture wearers. However, mechanical cleansing is not advisable for soft denture liners since it can damage resilient lining. Chemical cleansing by denture cleansers is first choice for denture plaque control. The solutions used for denture cleansing can be divided according to their chemical composition: alkaline peroxide, alkaline hypochlorite, acids, disinfectants and enzymes. Peroxide cleansers are the most commonly used denture cleansers. They are dispensed in powder or tablet forms, which become alkaline solutions of hydrogen peroxide when dissolved in water (9).

Although chemical cleansing has been considered an efficacious method to prevent Candida albicans invasion and denture plaque formation, some types of denture cleansers have been reported to cause significant deterioration of tissue conditioners in a relatively short time. A roughened surface facilitates colonization by microorganisms. Therefore denture cleansers used for plaque control of tissue conditioners should reduce microbial contamination and have a minimum effect on physical properties of liner (10).

The most common problems encountered while using soft denture liners are water sorption and solubility. In use, they are constantly bathed in saliva, and when out of the mouth, they are usually immersed either solution or denture cleansers or water for storage. During such immersion, soft lining materials undergo two responses: plasticizers and other soluble components are leached out and water or saliva is absorbed. So, an ideal processed soft liner should have no soluble components and low water sorption (11).

Total hundred and twenty specimens were made of dimension of 15 × 10 mm (according to ASTM: D -2240-64T). All the specimens were divided into six groups based on the relining materials and denture cleansers used and each group was tested at a time interval of day 1, day 7, day 30 and day 90. All the specimens were stored in artificial saliva throughout the study. Statistical analysis, showed that on day 1 both the test (AP and AE) and control (AW)acrylic liner groups showed no difference in hardness but there was significant (*P*<0.05) increase in hardness was noted between and within the all acrylic liner groups on day 7, day 30 and day 90. The highest mean hardness was observed in AP acrylic liner group followed by AE and AW acrylic liner group. Slight increase in hardness of all the silicon liner groups (SP, SE and SW) from day 1 to day 90 but a statistically significant (*P*<0.05) change was noted between and within the all silicon liner groups(SP, SE and SW) on day 7, day 30 and day 90. The highest mean hardness was observed in SP silicon liner group followed by SE and SW silicon liner group. There was an increase in mean surface roughness (Ra) from day 1 to day 90 of both test (AP and AE) and control (AW) acrylic liner groups but a statistically significant (*P*<0.05) increase in surface roughness was noted between and within the all acrylic liner on day 7, day 30 and day 90. Highest mean surface roughness was observed in AP acrylic liner group followed by AE and AW acrylic liner group. Very slight increase in mean surface roughness (Ra) of all the silicon liner groups from day 1 to day 90 and no significant (*P*>0.05) change in surface roughness was noted between and within the groups of the test (SP and SE) and control (AW) silicon liner groups on day 1.

Acrylic liner group showed higher surface hardness than silicon liner group when treated with cleanser P, cleanser E and cleanser W. These differences were statistically significant at all time intervals. Acrylic liner group showed higher surface roughness than silicon liner group when treated with cleanser P, cleanser E and cleanser W. These differences were statistically significant at all time intervals. Malherios-Segundo *et al* verified in their study that there was significant increase in hardness throughout their study in both groups (Control group-immersion in artificial saliva at 37°C and Experimental group – immersion in artificial saliva at 37°C combined with immersion in the cleanser for 5 min) for both the materials (Rigid liner–Kooliner and Soft liner- Elite soft). No significant alteration in surface roughness was caused by immersion in sodium perborate when compared to immersion in artificial saliva for the same time (7).

In our study, there is a significant increase in hardness and surface roughness in all the groups for both the material. However, there were more significant changes hardness in the acrylic liner group than silicone liner group. Hence, the above mentioned study is in favour of our study. Pahuja *et al* conducted a study which showed that silicone based soft denture liners had shown few changes in surface roughness for the first three months but highly significant changes were observed in surface hardness at 6 months, whereas, acrylicbased soft denture liners showed minor changes in surface hardness for 1 month, after which they showed significant increase in surface hardness at all intervals (9).

This study was in support of our study which showed significant increase in hardness and surface roughness in both the acrylic and silicone liners. However, increased hardness and surface roughness was more in the acrylic denture liners than in silicone liners. Brozek *et al* conducted a study to determine the effect of storage in disinfectants and artificial saliva on a series of commercial soft lining materials for dentures. They found that acrylic materials became less elastic on storage for 28 days whereas the silicone materials showed no changes (12).

Garcia *et al* demonstrated that the surface roughness was unaffected when resilient denture liner specimens were immersed in Polident solution. Whereas, in our study it was observed that surface roughness was increased for both acrylic and silicone liner groups when treated with Polident solution (15).

Denture cleansing affect the properties of the soft lining materials, reducing their elastomeric properties, acrylics was more adversely affected than silicone. These changes are associated with loss of various chemicals, including plasticizers and monomer, from the soft lining materials. These findings were in support of our study too. Tan *et al* mentioned in study that silicone denture liner when treated with perborate containing denture cleansers showed greater amounts of components could leach out from the liner leading to surface roughness. This study is in favour of our study where the denture cleansers used contained sodium perborate which might have resulted in the increase of surface roughness (13).

Mese *et al* mentioned in their study that silicone based resilient liner had significantly lower hardness values than the acrylic based resilient liner. Similar findings were observed where silicone liner group had lower surface hardness values than acrylic liner group (14).

Garcia *et al* demonstrated that the surface roughness was unaffected when resilient denture liner specimens were immersed in Polident solution. Whereas, in our study it was observed that surface roughness was increased for both acrylic and silicone liner groups when treated with Polident solution (15).

The increase in acrylic roughness might be related to the possible loss of soluble components, such as plasticizers, leaving empty spaces. Probably, with time, these empty spaces are responsible for the roughness, increase in size resulting in craters. The surfaces disturbances can also be related to porosity with lining. Air is entrapped during mixing and it appears that some of the cleansers cause the bubbles to increase in size- with some eventually reaching the surface. Such a roughened surface is likely to facilitate colonization by microorganisms (4).

Quantitative difference in cleanser formulations or pH of the solutions apparently influences the effect of cleanser on the properties of the liners. The use of hot water in combination with a cleanser may cause a more rapid deterioration (7).

The grade of surface porosity of soft liners varied depending on the immersion time and the combination of denture cleansers and soft liner. A previous study suggested that the oxygenation in strongly alkaline solutions is the damaging factor. They also suggested that peroxide content is one of the possible damaging factors and that other components or the pH of cleansers also affect the surface properties of soft liners (16).

However a direct comparison of the studies cannot be made because of different tests and different research protocols were used. It is difficult to relate the findings of the present study to other investigations because of the difference of sample size, type of resilient lining materials, duration of experiment, surface preparation and cleanser solutions used. It should be emphasized that the present study has limitations because only two of the many available soft lining materials and denture cleansers were evaluated and the study was performed in laboratory. Test conditions used for in-vitro study do not subject the materials to the aqueous environment, micro-organisms, temperature cycling, abrasion, material thickness and cycling loading. The properties of soft denture liners in the clinical situation still differ from laboratory testing. Further research in a well-controlled clinical trial will be fruitful.

## CONCLUSION

Within the limitations of the study following conclusions were drawn,

1. Significant increase in hardness was noted between and within all the acrylic liner groups at all time intervals. Maximum hardness was observed in AP acrylic liner group followed by AE and AW acrylic liner group.
2. There was a slight increase in hardness of all the silicon liner groups (SP, SE and SW) from day 1 to day 90. Significant difference was seen between the hardness of SP silicon liner group and SE silicon liner group but the hardness of SW silicon liner group remained relatively stable at all time intervals.
3. Initially the mean surface roughness was same for all the acrylic liner groups but as the time increased, surface roughness also increased, with maximum surface roughness was noted in AP acrylic liner group followed by AE and AW acrylic liner groups.
4. Effect of denture cleansers on silicon relining material showed slight increase in surface roughness of all the silicon liner groups (SP, SE and SW) from day 1 to day 90 no significant change in surface roughness was noted between and within the groups of the test (SP and SE) and control (AW).
5. Acrylic liner groups showed higher surface hardness than silicon liner groups at all time intervals. The hardness of acrylic liners and silicon liners increased both with time and also due to effect of denture cleansers.
6. Maximum surface hardness was noted by cleanser P (Polident) followed by cleanser E (Efferdent Plus) and cleanser W (Water) for both acrylic liner group and silicon liner group.
7. Maximum surface roughness was noted by cleanser P (Polident) followed by cleanser E (Efferdent Plus) and cleanser W (Water) for both acrylic liner group and silicon liner group.

This study is entirely laboratory based, however the most appropriate testing environment is the mouth and therefore long-term clinical studies of these materials is required.
